# Plasma Uric Acid Helps Predict Cognitive Impairment in Patients With Amyotrophic Lateral Sclerosis

**DOI:** 10.3389/fneur.2021.789840

**Published:** 2021-12-06

**Authors:** Jiahui Tang, Yuan Yang, Zhenxiang Gong, Zehui Li, Lifang Huang, Fengfei Ding, Mao Liu, Min Zhang

**Affiliations:** ^1^Department of Neurology, Tongji Hospital, Tongji Medical College, Huazhong University of Science and Technology, Wuhan, China; ^2^Department of Pharmacology, Shanghai Medical College, Fudan University, Shanghai, China; ^3^Department of Neurology, SUNY Downstate Medical Center, New York, NY, United States

**Keywords:** amyotrophic lateral sclerosis, cognitive impairment, biomarker, uric acid, ECAS

## Abstract

**Objective:** Uric acid as an antioxidant plays an important role in neurodegenerative disease. Our objective is to investigate the relationship between plasma uric acid and cognitive impairment in patients with amyotrophic lateral sclerosis (ALS).

**Methods:** In this cross-sectional study, 124 ALS patients were screened by the Edinburgh Cognitive and Behavioral Screen (ECAS) and classified according to the revised Strong's criteria. Additionally, based on total ECAS cut-off score patients were categorized into those with cognitive impairment (ALS-cie) and those without cognitive impairment (ALS-ncie), and clinical data and uric acid level were compared between the two groups. Parameters with significant differences were further included in a multivariate linear regression analysis with ECAS score as a dependent variable. Hold-out validation was performed to evaluate the fitness of regression model.

**Results:** Up to 60% of ALS patients showed cognitive or/and behavioral impairment. The ALS-cie group had lower education level (*p* < 0.001), older age at symptom onset (*p* = 0.001), older age at testing (*p* = 0.001), and lower plasma uric acid (*p* = 0.01). Multivariate analysis showed increased uric acid (β = 0.214, *p* = 0.01), lower age at testing (β = −0.378, *p* < 0.001), and higher education level (β = 0.424, *p* < 0.001) could predict higher ECAS score (*F* = 19.104, *R*^2^ = 0.381, *p* < 0.0001). Validation analysis showed that predicted ECAS score was significantly correlated with raw ECAS score in both the training set (*r*s = 0.621, *p* < 0.001) and the testing set (*r*s = 0.666, *p* < 0.001).

**Conclusions:** Cognitive impairment was a common feature in our Chinese ALS patients. Plasma uric acid might help evaluate the risk of cognitive impairment in ALS patients when combined with education level and age at testing.

## Introduction

Amyotrophic lateral sclerosis (ALS) is a fatal motor neuron disorder characterized by progressive loss of upper and lower motor neurons. Although ALS was initially considered only to involve the motor system, cognitive impairment has been increasingly recognized as one feature of ALS (1). While up to half of ALS patients had mild cognitive impairment, 15% of patients fulfilled the diagnostic criteria of frontotemporal dementia (FTD) (2, 3). The overlapping clinical (4), neuroimaging (5), neuropathological (6), and genetic features (7) suggested that ALS and FTD might constitute a disease spectrum. Since cognitive impairment could increase the caregiver burden and shorten survival (8), early detection of cognitive abnormalities in ALS patients is essential.

Diagnosis of cognitive impairment has been largely based on comprehensive neuropsychological tests (9), which could be limited by resource and time. Additionally, completion of those tests might be restricted by the physical disabilities of ALS patients. In contrast, blood biomarkers have the potential of being readily available. One study on hormonal peptides showed that plasma neuropeptide Y and leptin levels might be markers of cognitive changes measured by Addenbrooke's Cognitive Examination-Revised in ALS patients (10). Another study found significant differences in the plasma levels of 20 proteins by mass spectrometry analysis in 36 ALS patients with or without cognitive impairment using Addenbrooke's Cognitive Examination-III (11).

Oxidative stress was involved in pathological processes leading to neuronal damage in ALS (12). The neuroprotective role of plasma uric acid as an antioxidant has been studied widely in neurodegenerative diseases including ALS (13). Two case-control studies showed that ALS patients had significantly lower plasma uric acid than healthy controls, and the lower uric acid level was associated with faster disease progression (14, 15). Another two randomized clinical trials reported that ALS patients with a higher uric acid level at baseline had prolonged survival advantages (16, 17). Additionally, the uric acid level was reversely correlated with disease stages and significantly decreased during disease progression (18). Furthermore, a prospective study involving 319,617 participants found that uric acid level was inversely related to ALS risk in healthy individuals (19). However, the relationship between plasma uric acid level and cognitive impairment of ALS patients is still unknown.

In this study, we aimed to identify whether plasma uric acid could help evaluate cognitive impairment in Chinese ALS patients.

## Methods

### Participants

**Figure 1:**
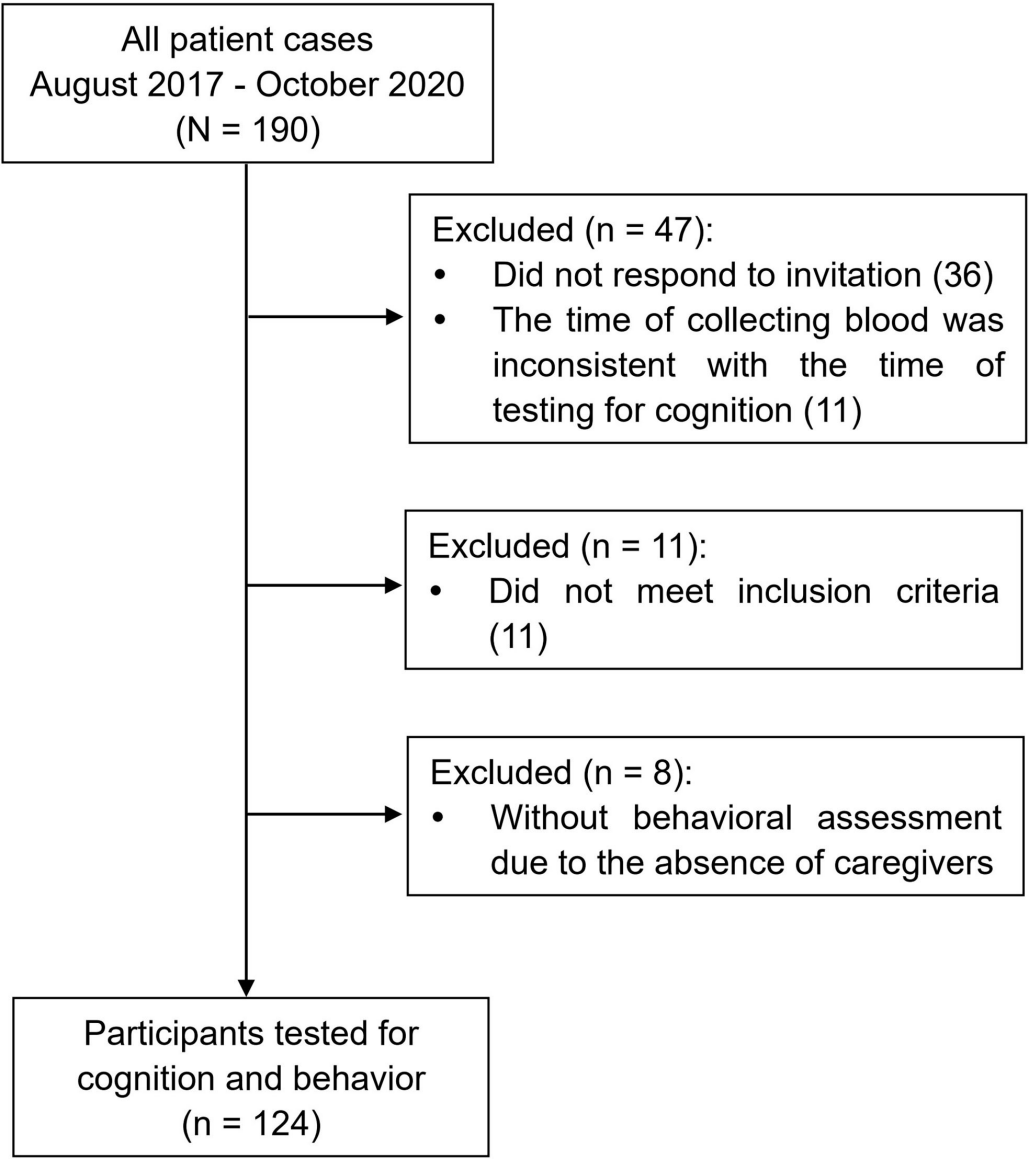
Flow chart reporting the enrollment of cases.

One hundred and ninety ALS patients (possible, probable, or definite ALS according to the El Escorial criteria) (20) who were admitted to Department of Neurology, Tongji Hospital in Wuhan between August 2017 and October 2020 were screened for this cross-sectional study. Exclusion criteria included other neurological disorders affecting cognitive function, such as stroke, traumatic brain injury, epilepsy; psychiatric disorders; major organ dysfunction; loss of both language and writing ability, and alcohol and drug abuse. Out of the 190 patients, 124 patients (65.3%) were finally included in this study (Figure 1). The study was approved by the Ethics Committee of Tongji Hospital, and all patients provided written informed consent to participate.

### Data Acquisition

Clinical information including sex, age, disease duration, education time, body mass index (BMI), and site of onset was collected. Patients were evaluated at their first visit by the Chinese version of Edinburgh Cognitive and Behavioral Screen (ECAS) (21), a screening tool for the comprehensive assessment of cognitive status of ALS patients. It consists of five cognitive domains, including language (28 points), executive functions (48 points), verbal fluency (24 points), memory (24 points), and visuospatial functions (12 points), which make up a total score of 136 points (21, 22). The cut-off total score (81.92 points) and subdomains scores were calculated as two standard deviations below the corresponding mean score of the healthy Chinese population (22). Additionally, ECAS includes a behavioral assessment of patients by caregivers (10 points), based on the key behavioral criteria of diagnosing behavioral variant FTD (21). It evaluates five behavioral domains including disinhibition (three points), apathy (one point), loss of sympathy (two points), perseveration (two points), and changes in eating behaviors (two points), as well as psychotic symptoms (three points). The severity of physical disability was measured by the amyotrophic lateral sclerosis functional rating scale-revised (ALSFRS-R), which evaluated bulbar, upper limb, lower limb, and respiratory function, with a higher score (total score 48 points) representing better physical function (23). Plasma uric acid level was measured via enzymatic colorimetric method using the Cobas c701 automatic analyzer (Roche) in the Department of Clinical Laboratory of Tongji Hospital.

### Classification

All patients were screened into two groups based on total ECAS score, i.e., the group with cognitive impairment (ALS-cie group, total score <81.92) and the group without cognitive impairment (ALS-ncie group, total score ≥81.92) (22). Additionally, patients with abnormality in at least one behavioral domain were considered to have behavioral abnormality.

Patients were further categorized into five classical groups according to the revised Strong's criteria: (1) ALS with cognitive impairment (ALSci), if patients had at least one abnormal symptom in the domain of language, executive function, or verbal fluency; (2) ALS with behavioral impairment (ALSbi), if patients had apathy or at least two other behavioral/psychotic symptoms; (3) ALS with combined cognitive and behavioral impairment (ALScbi), if patients met the criteria of both ALSci and ALSbi; (4) ALS-FTD, if patients had at least three abnormal symptoms of cognitive or behavior domain which were progressive; (5) ALS with normal cognition (ALSns), if patients did not meet any of the above mentioned criteria (24).

### Statistical Analysis

All statistical analyses were performed by SPSS statistical software (version 22.0). Normally distributed data were presented as the mean ± SD, while non-normally distributed data were showed as median (range). The distribution of categorical data was reported as frequencies and percentages. To compare clinical parameters and uric acid level between the two groups, the Chi-square test was used for categorical data, the independent *t*-test was used for normally distributed continuous data, and the Mann-Whitney-U-test for non-normally distributed continuous data. Significantly different parameters were further included in a multivariate linear regression analysis as independent variables, with the total ECAS score as a dependent variable. Education level was dichotomized into lower education (≤9 years) and higher education (>9 years) according to the median value. Moreover, the hold-out validation analysis was performed to assess the fitness of regression model by dividing patients randomly into two subsets, i.e., 80% as training set and 20% as testing set. After obtaining predicted ECAS scores through the regression model, correlations between predicted and raw ECAS scores were calculated in both training set and testing set. Additionally, correlations between plasma uric acid level and ECAS total/subdomain scores were analyzed by Pearson correlation or Spearman correlation in case of normally distributed data or non-normally distributed data, respectively. All statistical analyses were two-sided, and *p* < 0.05 was considered statistically significant.

## Results

### Clinical Characteristics

Of 124 ALS patients, 52 (41.9%) were women, and 72 (58.1%) were men. The mean age at onset was 53.6 ± 10.8 years, and the mean age at testing was 54.6 ± 10.9 years. The site of onset was limb onset in 93 patients (75%), followed by bulbar onset in 22 patients (17.7%), mixed onset in 8 patients (6.5%), and respiratory onset in 1 patient (0.8%). The median education time was 9 years, the median duration of illness was 11 months, the median BMI was 21.6, and the median ALSFRS-R score was 41 (Table 1).

**Table 1:** Clinical characteristics of ALS patients.

	**All patients**	**ALS-ncie**	**ALS-cie**	***P*-value**
	***n* =124**	***n* = 76**	***n* = 48**	
Male (%)	58	63	50	0.15^a^
Education (year)	9 (0–16)	11 (4–16)	7.5 (0–12)	<0.001^c^
Age at onset (year)	53.6 ± 10.8	51.1 ± 10.4	57.5 ± 10.4	0.001^b^
Age at testing (year)	54.6 ± 10.9	52.1 ± 10.5	58.6 ± 10.3	0.001^b^
Duration of illness (month)	11 (1–64)	10.5 (2–49)	11 (1–64)	0.41^c^
BMI (kg/m^2^)	21.6 (15.2–28.3)	21.6 (15.2–28.3)	21.6 (17.3–26.9)	0.93^c^
Site of onset: bulbar/limb/respiratory/mixed (%)	18/75/1/6	16/78/1/5	21/71/0/8	0.57^a^
ALSFRS-R score	41 (15–48)	41.5 (19–48)	40 (15–47)	0.57^c^
Uric acid (μmol/L)	310.4 ± 76.0	323.8 ± 77.1	289.3 ± 70.0	0.01^b^

*ALS-cie, amyotrophic lateral sclerosis with cognitive impairment; ALS-ncie, amyotrophic lateral sclerosis without cognitive impairment; BMI, body mass index; ALSFRS-R, amyotrophic lateral sclerosis functional rating scale-revised*.

a *χ^2^-test*.

b *Independent t-test with values presented as mean ± SD*.

c *Mann-Whitney-U-test with values presented as median (range)*.

Patients in the ALS-cie group had shorter education time (7.5 vs. 11 years, *p* < 0.001), older age at symptom onset (57.5 ± 10.4 vs. 51.1 ± 10.4 years, *p* = 0.001), older age at testing (58.6 ± 10.3 vs. 52.1 ± 10.5 years, *p* = 0.001), and lower plasma uric acid level (289.3 ± 70.0 vs. 323.8 ± 77.1, *p* = 0.01) than those in the ALS-ncie group. Sex ratio, disease duration, site of onset, BMI values, and ALSFRS-R score did not differ between the two groups (Table 1).

### Cognitive and Behavioral Status of ALS Patients

**Figure 2:**
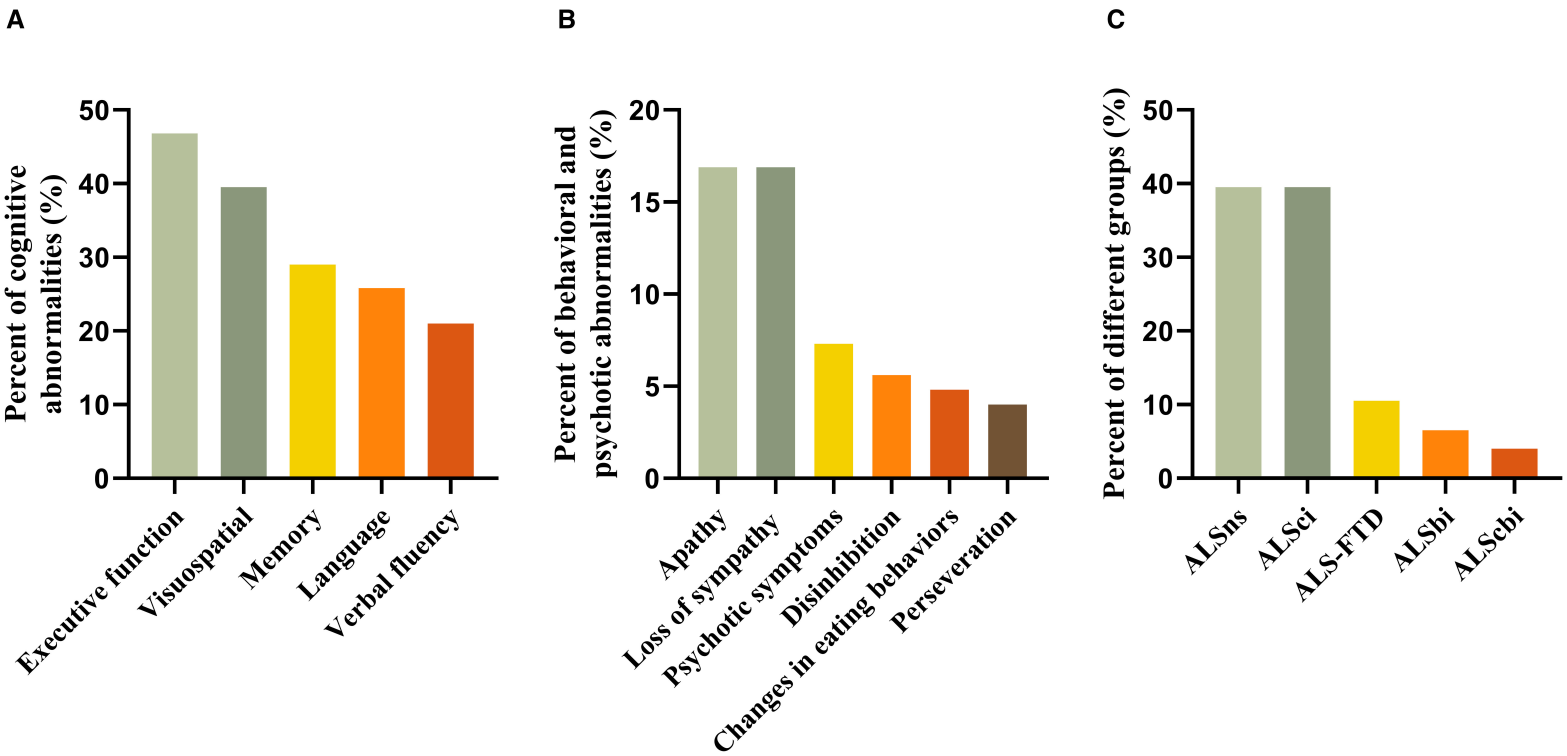
**(A–C)** Frequency of cognitive and behavioral subdomain impairment as well as ALS grouping according to the revised Strong's criteria.

Forty-eight (38.7%) patients had abnormal ECAS total scores based on a cut-off value of 81.92. Of five cognitive domains, executive impairment was the most frequent (46.8%), followed by visuospatial (39.5%) and memory (29.0%) impairment. Around one-fifth of patients had language (25.8%) and verbal fluency (21.0%) impairment (Figure 2A).

Behavioral and psychotic abnormalities were observed in 39 (31.5%) of patients, with 21 (16.9%) showing abnormalities in one, 10 (8.1%) in two, and 7 (5.6%) in three or more domains. Apathy (16.9%) and loss of sympathy (16.9%) were the most common, followed by psychotic symptoms (7.3%), disinhibition (5.6%), changes in eating behaviors (4.8%), and perseveration (4.0%) (Figure 2B).

According to the revised Strong's criteria, 49 patients (39.5%) had ALSns, 49 (39.5%) patients had ALSci, 13 (10.5%) had ALS-FTD, 8 (6.5%) had ALSbi, and 5 (4.0%) had ALScbi (Figure 2C).

### The Relationship Between Plasma Uric Acid and Cognitive Subdomains

Plasma uric acid level was weakly associated with scores of language (*r*s = 0.268, *p* = 0.003) and executive domain (*r*s = 0.179, *p* = 0.047). No significant correlations between uric acid and verbal fluency, memory, and visuospatial functions were identified (Table 2).

**Table 2:** The relationship between plasma uric acid and cognitive domains.

**Cognitive subdomains**	***r*s**	***P*-value**
Language	0.268	0.003
Fluency	0.068	0.456
Executive functions	0.179	0.047
Memory	0.147	0.103
Visuospatial functions	0.14	0.121

*rs, Spearman's relational coefficient*.

### Multivariate Regression Analysis and Model Validation

Education level, age at testing (age at onset was not selected due to their collinearity relationship), and plasma uric acid level were included in a lineal regression analysis. Increased uric acid (β = 0.214, *p* = 0.01), lower age at testing (β = −0.378, *p* < 0.001), and higher education level (β = 0.424, *p* < 0.001) were significant predictors of higher ECAS score (*F* = 19.104, *R*^2^ = 0.381, *p* < 0.0001) (Table 3). Validation analysis showed predicted ECAS scores were significantly correlated with raw ECAS scores in both the training set (*r*s = 0.621, *p* < 0.001) and the testing set (*r*s = 0.666, *p* < 0.001), indicating reasonable model fitness.

**Table 3:** Multivariate model of the total ECAS score.

**Variable**	***B***	**β**	**95% CI**	***P*-value**
Education level	21.324	0.424	13.145 to 29.503	<0.001
Age at testing, years	−0.854	−0.378	−1.207 to −0.483	<0.001
Uric acid, μmol/L	0.07	0.214	0.017 to 0.123	0.010

*B, regression coefficient; β, standardized regression coefficient; CI, confidence interval*.

## Discussion

Our findings for the first time showed that cognitive or behavioral dysfunction occurred in around 60% of Chinese ALS patients according to the revised Strong's criteria (24). Among all cognitive and behavioral subdomains, executive dysfunction was the most common subtype of cognitive impairment, while apathy and loss of sympathy were the main subtypes of behavioral abnormality. Importantly, we found that low plasma uric acid could help predict cognitive impairment in ALS patients along with aging and low educational level.

While the percentages of ALSbi and ALScbi in our ALS cohort were similar to previously reported data, ALSci (39.5% vs. around 16%) was more than doubled and ALS-FTD (10.5% vs. around 20%) was around half compared to the Italian populations (mean age 66) (25, 26). Another study showed the percentage of overall cognitive impairment (36 vs. 22%, ECAS total cut-off score) as well as executive dysfunction (47 vs. 20%) is higher among the ALS patients from China (mean age 55) than those from Germany (mean age 60), while the authors stated that the language used in ECAS questionnaire could not fully account for the differences (27). Interestingly, the percentage of cognitive dysfunction from our ALS cohort (mean age 55) based on the ECAS criteria was highly consistent with the data of Chinese patients recruited in another major academic center (22). Although other factors including educational level might influence ECAS performance, these findings support that race related differences in neurobiological networks might play a role in the different performances of Chinese and Caucasian populations (27). While executive dysfunction was the most common cognitive change in ALS patients consistent with previous studies (22), our study found that cognitive domains including memory and visual space were also frequently affected (28, 29). While some studies proposed that memory impairment was due to the failure of encoding secondary to executive impairment (30), a recent finding suggested that memory impairment was a primary dysfunction due to temporal lobe involvement in ALS patients (31). Similarly, poor visuospatial performance might be associated with temporal lobe involvement (32).

Our ALS patients were significantly younger and had lower bulbar onset percentage (18 vs. 33%) than the Italian cohort (53.6 vs. 65.5 years), which could contribute to the lower prevalence of ALS-FTD (26). In addition, C9orf72 gene mutation was strongly associated with ALS-FTD (26). The lower percentage of ALS-FTD in our ALS cohort could be partly attributed to the significantly lower prevalence of C9orf72 gene mutation in the Chinese than the Caucasian populations (33). In line with previous studies (34–36), apathy and loss of sympathy were common behavioral changes in ALS patients.

Our study showed lower plasma uric acid level was an independent predictor of cognitive impairment. Additionally, plasma uric acid level was positively, although weakly, correlated with executive and language domains of ECAS. Uric acid is the end-product of purine metabolism (37). Hyperuricemia is associate with gout with the deposition of monosodium urate in joints (38). Additionally, uric acid has been shown to act as a major antioxidant in the human body by scavenging reactive oxygen species (ROS) and peroxynitrite (39) and inhibiting iron-mediated oxidation through chelation (40). A prospective study involving 4,618 participants aged 55 years or older found that higher plasma uric acid level was associated with better cognitive function and a decreased risk of dementia after adjusting for cardiovascular risk factors (41). Another study conducted on 111 patients with tauopathies, including FTD, Alzheimer's disease (AD), and progressive supranuclear palsy, found lower plasma uric acid in patients compared to healthy controls and demonstrated that plasma uric acid level was inversely associated with risk of tauopathies independent from age and gender (42). One previous study using astroglial cultures showed that uric acid could boost glutathione production through astrocytic molecular pathways (43) and might play a protective role in reducing oxidative stress in tauopathies (44).

The most frequently identified pathology in the ALS-frontotemporal spectrum disorder is the accumulation of transactive response DNA-binding protein 43 (TDP-43) in the cytoplasm of neurons (6). One study found a significant difference in the severity of TDP-43 pathology between ALS-FTD and non-demented ALS patients (45). Another study further confirmed that ALS-specific cognitive impairment of verbal fluency, language, and executive function based on ECAS was highly correlated with TDP-43 pathology in the corresponding functional lobal areas in ALS patients without clinically diagnosed dementia (46). A post-mortem ALS study found that TDP-43 aggregation showed temporal progression patterns across different disease stages with the TDP-43 pathology expanding from the motor cortex to the prefrontal and temporal lobe (47).

Of note, mitochondria dysfunction and oxidative stress have been reported to play an important role in TDP-43 pathology of cellular and animal models (48, 49). Basic *in vivo* and *in vitro* studies showed that TDP-43 could induce mitochondrial ROS production and negatively affect neuronal survival and function (50). The nuclear factor erythroid 2-related factor 2 (Nrf2) was a main regulatory factor in preventing the accumulation of ROS and reducing oxidative stress (51). Overexpression of Nrf2 in astrocytes could delay symptom onset and prolong survival in ALS mouse models (52). Protective effects of uric acid on motor neurons could be exerted by activating Nrf2 expression in ALS models, leading to increased glutathione, and decreased oxidative damage (53). Furthermore, deficiency of Nrf2 expression could aggravate the impairment of recognition memory in a neuroinflammatory mouse model (54), while induction of Nrf2 expression alleviated cognitive impairment in an AD mouse model (55). We thus postulate that higher plasma uric acid level could help attenuate ROS production and oxidative stress in brain areas critical to maintaining normal cognition in ALS patients. One study proposed that lower uric acid levels in ALS patients might be due to malnutrition attributed to the bulbar onset and longer disease duration (56). Our study showed no difference in BMI values, disease duration, ALSFRS-R score, and bulbar onset between ALS-ncie and ALS-cie, thus not supporting a correlation between nutrition and uric acid level in our ALS patients. Inosine, the urate precursor, was proven to increase serum uric acid level safely and tolerably when administered orally or via feeding tubes in a small pilot trial of ALS patients (57). It would be interesting to explore whether inosine helps prevent or mitigate cognitive decline in ALS patients in future longitudinal studies.

The current study had several limitations. The cognitive and behavioral status of our patients was only evaluated by ECAS, thus not fully fulfilling the technical requirements of the revised Strong's criteria for accurate classification, for example, primary progressive aphasia and loss of insight could not be evaluated in a standardized way. Our study has a small sample size due to the low incidence of ALS and all participants were recruited in a single tertiary center, making referral bias possible. In addition, our study had a cross-section design and plasma uric acid was not dynamically measured, weakening its predictive strength. Furthermore, genetic features including C9orf72 mutation status that could affect the cognitive and behavioral status of ALS patients (26, 58) were not obtained due to limited resources. Of note, age was one of the major factors affecting cognitive function. It had been showed that hypertension, diabetes, cardiovascular disease, smoking, systemic inflammation, and stress were associated with cognitive impairment during the aging process (59), and these factors were not included and comprehensively evaluated in our regression model. Lastly, we did not have the neuroimaging data to explore potential correlation between cognitive/behavioral status and specific brain areas' structural or functional changes (47).

## Conclusions

Cognitive impairment was a common feature in the Chinese ALS patients. Decreased plasma uric acid was an independent risk factor of cognitive dysfunction in ALS patients apart from aging and low educational level. Longitudinal studies on the dynamic changes in plasma uric acid and cognitive status of ALS patients could help further clarify their relationship during disease progression which might provide potential therapeutic options.

## Data Availability Statement

The raw data supporting the conclusions of this article will be made available by the authors, without undue reservation.

## Ethics Statement

The studies involving human participants were reviewed and approved by the Ethics Committee of Tongji Hospital. The patients/participants provided their written informed consent to participate in this study.

## Author Contributions

JT, MZ, and FD contributed to the study design. JT, YY, ZG, ZL, and LH contributed to data acquisition. JT and ML contributed to data interpretation and statistical analysis. JT drafted the manuscript. ML and MZ revised the manuscript. All authors contributed to the article and approved the submitted version.

## Funding

This study was supported by the clinical research program of Bethune Charitable Foundation.

## Conflict of Interest

The authors declare that the research was conducted in the absence of any commercial or financial relationships that could be construed as a potential conflict of interest.

## Publisher's Note

All claims expressed in this article are solely those of the authors and do not necessarily represent those of their affiliated organizations, or those of the publisher, the editors and the reviewers. Any product that may be evaluated in this article, or claim that may be made by its manufacturer, is not guaranteed or endorsed by the publisher.
